# Fingolimod Inhibits Inflammation but Exacerbates Brain Edema in the Acute Phases of Cerebral Ischemia in Diabetic Mice

**DOI:** 10.3389/fnins.2020.00842

**Published:** 2020-08-11

**Authors:** Wanlu Li, Tingting He, Lu Jiang, Rubing Shi, Yaying Song, Muyassar Mamtilahun, Yuanyuan Ma, Zhijun Zhang, Yaohui Tang, Guo-Yuan Yang, Yongting Wang

**Affiliations:** ^1^School of Biomedical Engineering, Med-X Research Institute, Shanghai Jiao Tong University, Shanghai, China; ^2^Department of Neurology, Ruijin Hospital, School of Medicine, Shanghai Jiao Tong University, Shanghai, China; ^3^Department of Neurology, Zhongshan Hospital, Fudan University, Shanghai, China; ^4^Department of Neurology, Renji Hospital, School of Medicine, Shanghai Jiao Tong University, Shanghai, China

**Keywords:** diabetic stroke, diabetes mellitus, fingolimod, edema, inflammation

## Abstract

**Background and Purpose:** Diabetes mellitus increases stroke incidence and mortality and hampers functional recovery after stroke. Fingolimod has been shown to improve neurofunctional recovery and reduce brain infarction after ischemic injury in mice without comorbidities. In this work, we investigated the effects of fingolimod in diabetic mice after transient middle cerebral artery occlusion (tMCAO).

**Methods:** Hyperglycemia was induced by a single bolus streptozotocin injection. Adult male ICR mice (*n* = 86) underwent 1-h tMCAO surgery and received intraperitoneal injection of fingolimod (1 mg/kg) or vehicle immediately after reperfusion. Clark neurological score, brain infarction and edema, blood–brain barrier (BBB) integrity, apoptosis, and inflammation were evaluated at 24 h after tMCAO.

**Results:** Fingolimod treatment reduced the number of infiltrated inflammatory cells and lowered the mRNA level of *Tnf*α. It also increased the ratio of Bcl-2/Bax. However, fingolimod significantly aggravated brain edema and reduced the expression levels of tight junction proteins ZO-1 and Occludin. The negative impacts of fingolimod on BBB integrity outweighed its beneficial effects in anti-inflammation, which resulted in the lack of improvement in endpoint outcomes at 24 h after tMCAO.

**Conclusion:** Caution should be taken in considering the acute treatment using fingolimod for ischemic stroke with diabetes comorbidity.

## Introduction

Diabetes increases the incidence of stroke and post-stroke mortality (Barrett-Connor and Khaw, 1988). Compared to non-diabetic stroke patients, diabetic stroke patients present exacerbated brain damage and worsened functional recovery. The aggravated damage and hampered recovery following stroke with diabetes comorbidity resulted from many interconnected processes, including inflammation, BBB disruption, abnormal vascular function, reduced neurogenesis, and suppressed oligodendrogenesis (Hawkins et al., 2007; Tureyen et al., 2011; Prakash et al., 2012; Tan et al., 2015; Yatomi et al., 2015; Zhang et al., 2016).

Fingolimod (FTY720) is approved by the Food and Drug Administration (FDA) of the US for the treatment of multiple sclerosis. Fingolimod is phosphorylated by sphingosine kinase *in vivo*, and the phosphorylated fingolimod is an analog of S1P, an extracellular lipid that binds with cognate G protein-coupled receptors (Billich et al., 2003). Phosphorylated fingolimod prevents lymphocytes egressing from lymphoid tissues by activating S1PR1 on lymphocytes to facilitate its internalization, consequently reducing lymphocyte infiltration into the brain (Brinkmann et al., 2002; Graler and Goetzl, 2004; Matloubian et al., 2004). S1P receptors are also expressed on many types of cells in the CNS, including neurons, endothelial cells, astrocytes, microglia, and neural stem cells (Mutoh and Chun, 2008; Mutoh et al., 2012).

Growing works support that fingolimod is a protective agent after ischemic stroke in animal models without diabetes comorbidity. A clinical trial showed that treatment with three doses of fingolimod upon acute onset reduced secondary lesion enlargement and resulted in better clinical outcomes during the acute phase and 3-month follow-up (Fu et al., 2014). Studies found that fingolimod reduced brain infarct volume and improved neurological function at 24 h after stroke in normal mice (Czech et al., 2009). Acute treatments with fingolimod were also found to promote functional recovery at 7 or 14 days after stroke (Wei et al., 2011; Brunkhorst et al., 2013; Nazari et al., 2016; Schuhmann et al., 2016). However, inconsistent observations have been reported regarding the effect of fingolimod on brain edema after stroke (Liesz et al., 2011; Wei et al., 2011). It has been noted that patients with diabetes are likely at higher risk for the development of macular edema with fingolimod (Singer, 2013). Patients with MS and diabetes mellitus were excluded from the phase III clinical trials, so the true incidence of macular edema in patients with diabetes who are receiving fingolimod is unknown. Since diabetes is a common comorbidity of stroke, it is important to further understand the risk and benefit of using fingolimod in the acute phase after stroke with diabetes comorbidity.

In this study, we investigated the effects of fingolimod in treating cerebral ischemic injury with diabetes comorbidity within 24 h. We observed the beneficial effects of fingolimod in reducing lymphocyte infiltration. Unfortunately, fingolimod treatment in the acute phase of diabetic stroke resulted in significantly aggravated brain edema that overshadows its beneficial anti-inflammatory effects.

## Methods

### Experimental Design

**FIGURE 1:**
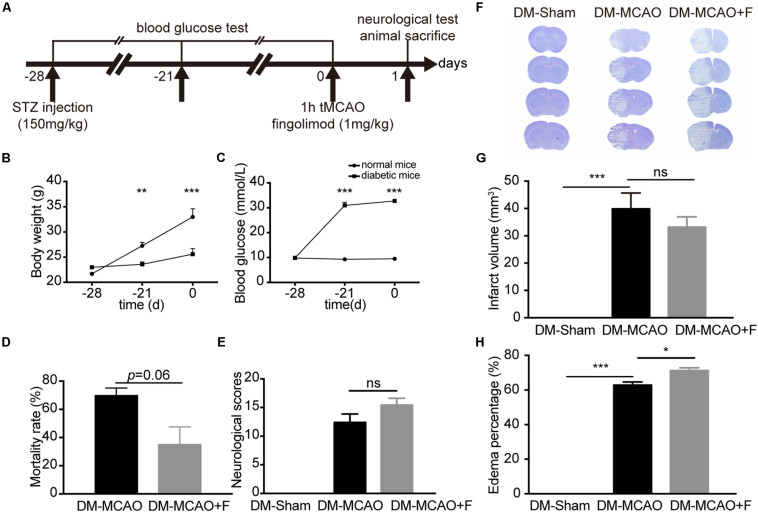
Fingolimod aggravated brain edema and did not improve the neurological outcome of diabetic stroke mice at 24 h. **(A)**, The experimental scheme. **(B,C)**, Line charts showing the changes in body weight **(B)** and blood glucose levels **(C)** of mice during the experiment. Detailed body weight and glucose data are presented in Supplementary Table S1. **(D)**, Bar graph summarizing the mortality rate at 24 h after tMCAO in DM + MCAO (*n* = 32, 10 animals survived) and DM + MCAO + F groups (*n* = 38, 27 animals survived), **(E)**, Neurological scores in DM-Sham (*n* = 6), DM + MCAO (*n* = 6) and DM + MCAO + F (*n* = 12) groups. **(F–H)**, Representative photos of coronal sections stained with cresyl violet at 24 h after tMCAO **(F)**. Quantification of brain infarct volume **(G)** and edema percentage **(H)** based on the result of cresyl violet staining [*n* (Sham) = 7, *n* (DM + MCAO) = 6, *n* (DM + MCAO + F) = 9]. Data are presented as mean ± SEM, *p < 0.05, **p < 0.01, ***p < 0.001. STZ streptozotocin; tMCAO, transient middle cerebral artery occlusion; DM-sham, diabetes mellitus sham control; DM + MCAO, MCAO carried out in diabetic mice treated with saline; DM + MCAO + F, DM + MCAO mice treated with fingolimod.

The animal experimental procedures were performed in accordance with the protocols approved by the Institutional Animal Care and Use Committee of Shanghai Jiao Tong University, China. Adult (10–12 weeks old) male Institute of Cancer Research (ICR) mice (*n* = 86) weighing 20–25 g were housed in SPF housing under a 12-h light-dark cycle for 2 weeks prior to the experiment. Age and sex differences were not investigated in this pilot study. The experimental design is presented in Figure 1A.

### Induction of Hyperglycemia and Transient Middle Cerebral Artery Occlusion Surgery

Hyperglycemia was induced by intraperitoneally injecting a single dose of STZ (150 mg/kg) to 8-h-fasted animals as described (Geng et al., 2017). Transient middle cerebral artery occlusion was performed as previously described (He et al., 2017). Cerebral blood flow (CBF) was measured with laser Doppler flowmetry (Moor Instruments, Devon, United Kingdom) before surgery, after occlusion, and after reperfusion to ensure accurate model control. Successful occlusion was defined by the decrease of surface CBF to less than 15% of the baseline. Successful reperfusion is defined by the restoration of surface CBF to 80% of the baseline after suture withdraw. Animals presented with unsuccessful occlusion or reperfusion were excluded from further study. Further details are presented in the Supplementary Material.

### Fingolimod Treatment

Fingolimod (Sigma-Aldrich) was dissolved in normal saline to 0.1 mg/ml and stored in aliquots at −20°C. Prior to use, the fingolimod solution was thawed at room temperature. For treatment, fingolimod was injected intraperitoneally immediately after reperfusion at 1 mg/kg dose. This dose was used in previously reported stroke animal studies by several other research groups (Czech et al., 2009; Liesz et al., 2011; Wei et al., 2011; Schuhmann et al., 2016). Animals were randomly assigned into three different groups, with 10 in the sham group, 32 in the normal saline vehicle group (DM + MCAO), and 38 in the fingolimod treatment group (DM + MCAO + F). The surgery was carried out in 5 days with 16 surgeries per day. In the first three days of surgery, odd numbered animals were assigned to DM + MCAO saline control group and even numbered animals were assigned to DM + MCAO + F group, with the exception of the last two animals, which were assigned to the Sham group. Animals in the sham group underwent hyperglycemia induction, anesthesia, midline incision on the neck, and isolation of the left common carotid artery (CCA), the external carotid artery (ECA) and the internal carotid artery (ICA) without insertion of a suture to occlude the MCA. In the last two days of surgery, two more animals were assigned into the DM + MCAO + F group, due to slightly larger standard deviation of the neurological outcome observed within the DM + MCAO + F group in the first three days.

### Neurological Behavioral Tests and the Assessment of Brain Lesion Size

Neurological outcome was assessed by an investigator blinded to the treatment assignment and surgery status. Neurological function at 24 h was assessed using the Clark neurological score (0–28) (Cheung, 2000). Clark neurological score test evaluates behavioral deficits according to the performance on body symmetry, gait, climbing, circling, front limb behavior, compulsory circling, and whisker response. Worse behavioral performance correlates with a higher score in the neurological score test. Brain infarct volume and edema were assessed based on cresyl violet staining (Huang et al., 2013; Ren et al., 2014). Detailed methods and equations for calculation are presented in the Supplementary Material.

### Immunohistochemistry

Immunohistochemistry against myeloperoxidase (MPO), tight junction protein 1 (ZO-1), and occludin were carried out using established protocols (Geng et al., 2017). In brief, brain frozen sections were permeabilized with 0.3% Triton X-100 (Sigma-Aldrich) for 10 min. After blocking with bovine serum albumin (BSA) (Yeasen, Shanghai, China), brain sections were incubated with primary antibodies overnight at 4°C followed by washing steps and then incubation with secondary antibodies for 1 h at room temperature. The primary antibodies were MPO (R&D System, MN, 1:200), ZO-1 (Invitrogen, Waltham, MA, United States 1:100), Occludin (Invitrogen, 1:100). The brain section images were obtained using a confocal microscope (Leica, Germany) and were analyzed by the NIH ImageJ software. For integrated optical density (IOD) of MPO staining, micrographs of 3 fields in the peri-infarct cortex region were captured in each section. These three fields were depicted with yellow frames in Figure 4A. Three coronal brain sections spaced 200 μm apart were sampled for every mouse. The gap formation of tight junction proteins ZO-1 and Occludin was quantified using the percentage of the gap distance of the total CD31-positive microvessel length.

Immunoglobulin G (IgG) leakage was examined as previously described (Liu et al., 2014). Brain cryosections were incubated with 0.3% H_2_O_2_ in methanol for 30 min followed by permeabilization with 0.1% Triton X-100 for 30 min. The sections were then blocked with 5% BSA for 30 min and then incubated with biotinylated universal antibody (Vector, Burlingame, CA, United States) for 30 min. After washing steps, the sections were incubated with Vectastain ABC reagent (Vector) for 30 min. The sections were developed using a DAB staining kit. Three fields in the peri-infarct region were photographed for each section, and three sections spaced 200 μm apart were analyzed for each brain. IOD of IgG was calculated using Image Pro Plus 6.0 software (Media Cybernetics, United States).

Terminal-deoxynucleotidyl transferase mediated nick end label staining was carried out following the manufacture protocol of a Cell Death Detection Kit (Roche, Basel, Switzerland) and quantified as previously described (Tang T. et al., 2014). Briefly, brain cryosections were permeabilized with 0.1% Triton X-100 for 2 min at 4°C and then incubated with TUNEL reaction mixture for 1 h at 37°C. Micrographs of three fields in the peri-infarct region were captured by the confocal microscope in each section, and three sections spaced 200 μm apart were analyzed for every brain to calculate TUNEL positive cells.

### Western Blot Analysis and Real-Time Quantitative PCR

Mice were sacrificed under deep anesthesia at 24 h after tMCAO. Brains were quickly removed and cut into four 2-mm-thick sections around the infarction region. The second section of the ipsilateral hemisphere was used to extract proteins. Western blot was performed as previously described (Tang Y. et al., 2014). The third section of the ipsilateral hemisphere was used to extract RNA. The cortex and striatum in each hemisphere were separated. RT qPCR procedure was carried out as previously described (Chang et al., 2017). Further details are presented in the Supplementary Material.

### Statistical Analysis

DM-Sham and DM + MCAO mice were used as controls. All results were presented as mean ± standard error of mean (SEM). All statistical analysis was carried out with SPSS v21.0 (SPSS Inc., Chicago, IL, United States) by one-way ANOVA followed by Two-tailed unpaired Student’s *t*-test. Dunnett’s test was used to compare many experimental treatments each to a single control following one-way ANOVA. A value of *p* < 0.05 was considered statistically significant.

## Results

### Acute Treatment With Fingolimod Failed to Improve Endpoint Outcomes at 24 h After tMCAO in Diabetic Mice

Transient middle cerebral artery occlusion surgery was performed at 28 days after STZ injection. STZ (150 mg/kg) injection was sufficient to raise the blood glucose to the hyperglycemic level (>16.7 mmol/L) and maintain hyperglycemia from 7 to 28 days after STZ injection (Figure 1C). Elevated blood glucose was accompanied by slower weight gain compared to control mice (Figure 1B).

Fingolimod treated group presented a trend of lower mortality rate at 24 h, albeit statistically insignificant (DM + MCAO + F vs. DM + MCAO, 35 ±12.58 vs. 69.83 ±5.247%, *p* = 0.06) (Figure 1D). No significant difference in neurological score (DM + MCAO vs. DM + MCAO + F, 12.40 ±3.29 vs. 15.42 ±4.12) (Figure 1E) or brain infarction volume or brain infarction volume (DM + MCAO vs. DM + MCAO + F, 40.7 ± 8.90 mm^3^ vs. 34.4 ± 6.97 mm^3^) (Figures 1F,G) was observed.

### Acute Treatment With Fingolimod Further Compromised BBB Integrity and Exacerbated Brain Edema at 24 h After tMCAO

**FIGURE 2:**
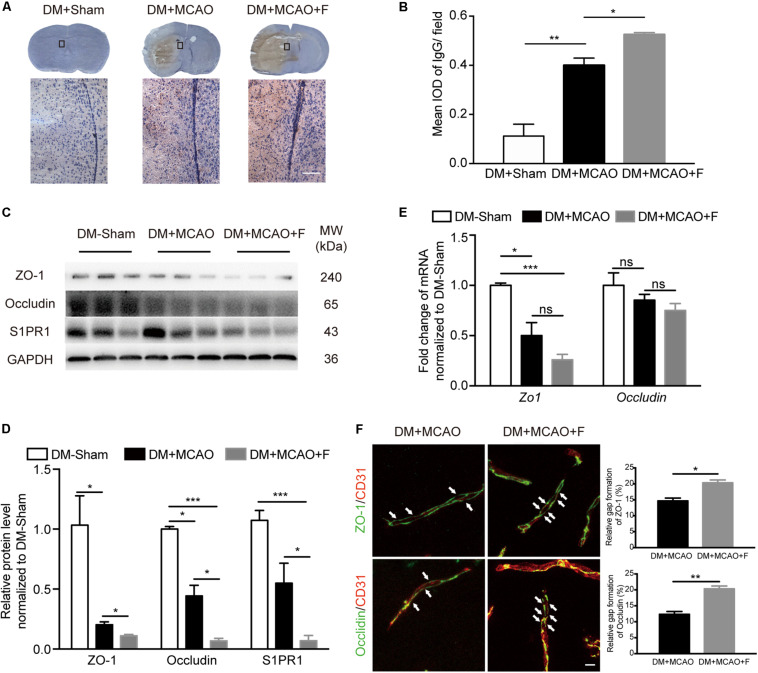
Fingolimod exacerbated blood–brain barrier (BBB) impairment at 24 h after tMCAO. **(A)**, Images of DAB immunostaining against immunoglobulin G (IgG) and H&E staining of coronal sections, the bottom panel shows the higher-magnification images of the boxed areas. Scale bar = 100 μm. **(B)**, Integrated optical density (IOD) quantification of IgG^+^ signal in the ipsilateral hemisphere at 24 h after tMCAO (*n* = 4/group). **(C,D),** Western blot results of ZO-1, Occludin, and Sphingosine-1-phosphate receptor 1 (S1PR1). Quantification of protein levels relative to GAPDH showed in bar chart [*n* (Sham) = 3, *n* (DM + MCAO) = 3, *n* (DM + MCAO + F) = 6]. MW: molecular weight. **(E)**, Bar graph showing relative mRNA expression of *Zo1* and *Occludin* compared with DM-Sham group [*n* (Sham) = 3, *n* (DM + MCAO) = 3, *n* (DM + MCAO + F) = 6]. **(F)**, Micrographs of immunostaining of tight junction proteins, ZO-1 and Occludin, and endothelial marker CD31. Arrows indicating the gaps of these junction proteins on the vessels in the ipsilateral hemispheres. Scale bar, 5 μm. Bar graph showing the relative gap of Zo1 or Occludin formation quantified with the micrographs. [*n* (DM + MCAO) = 3, *n* (DM + MCAO + F) = 3]. Data are presented as mean ± SEM, *p < 0.05, **p < 0.01, ***p < 0.001.

The degree of brain edema was higher in the fingolimod-treated group compared with the normal saline group (Figure 1H; DM + MCAO + F vs. DM + MCAO, 71.22 ±1.623 vs. 62.81 ±1.818%, *p* < 0.05). BBB integrity was assessed using IgG staining and the expression of tight junction proteins, ZO-1 and Occludin, as well as the protein level of S1PR1, a protein associated with BBB permeability. The result showed that IgG leakage was significantly increased in fingolimod-treated mice compared with control mice (Figures 2A,B; DM + MCAO + F vs. DM + MCAO, 0.5248 ±0.0083 vs. 0.4 ±0.02952, *p* < 0.05). ZO-1 and Occludin levels were reduced in both protein (Figures 2C,D; ZO-1, DM + MCAO + F vs. DM + MCAO, 0.111 ±0.010 vs. 0.203 ±0.089, *p* < 0.05. Occludin, DM + MCAO + F vs. DM + MCAO, 0.068 ±0.021 vs. 0.444 ±0.089, *p* < 0.05) and mRNA (Figure 2E) levels. Fingolimod also reduced the expression level of S1PR1 protein (Figure 2C,D). Double immunostaining of CD31/ZO-1 and CD31/Occludin showed higher degree of discontinuous staining of ZO-1 and Occludin in CD31^+^ microvessel in fingolimod-treated mice (Figure 2F, indicated by arrows), suggesting increased disruption of BBB integrity.

### Acute Fingolimod Treatment Increased Bcl-2/Bax Ratio but Did Not Reduce the Number of TUNEL+ Cells at 24 h After tMCAO

**FIGURE 3:**
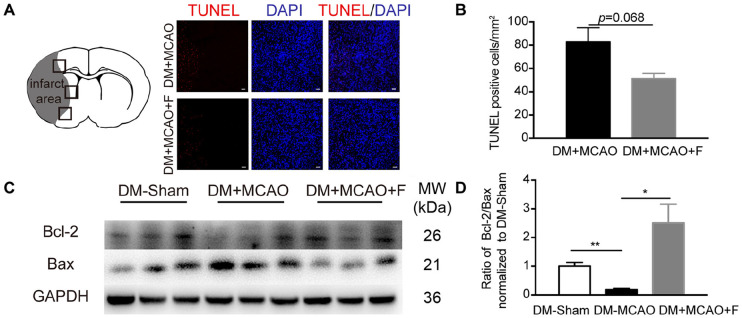
Fingolimod increased Bcl-2/Bax ratio but did not reduce the number of TUNEL + cells at 24 h after tMCAO. **(A)**, Micrographs of TUNEL staining in the peri-infarct region of DM + MCAO and DM + MCAO + F groups. Scale bar, 50 μm. Left panel showing the sketch of the brain section, boxes in it illustrating fields we are sampling in. **(B)**, Bar graph is the quantification of the number of TUNEL^+^ cells in DM + MCAO and DM + MCAO + F groups (*n* = 3/group). **(C)**, Western blot results of apoptotic factors Bcl-2 and Bax. **(D)**, Bar graph is the quantification of Bcl-2/Bax ratio based on Western blot data [*n* (Sham) = 3, *n* (DM + MCAO) = 3, *n* (DM + MCAO + F) = 6]. MW, molecular weight. Data are presented as mean ± SEM, *p < 0.05, **p < 0.01.

The number of apoptotic cells (TUNEL^+^) is not statistically different between the fingolimod-treated group and the saline control group (Figures 3A,B; DM + MCAO + F vs. DM + MCAO, 51.32 ±4.522 vs. 83.01 ±11.99 cells, *p* = 0.068). Western blot revealed that the ratio of anti-apoptotic protein Bcl-2 to pro-apoptotic protein Bax was higher in fingolimod-treated group (Figures 3C,D; DM + MCAO + F vs. DM + MCAO, 0.5244 ±0.1378 vs. 0.03705 ±0.01126, *p* < 0.05).

### Acute Fingolimod Treatment Inhibited Neutrophil Infiltration and Reduced the mRNA Level of *Tnf*α at 24 h After tMCAO

**FIGURE 4:**
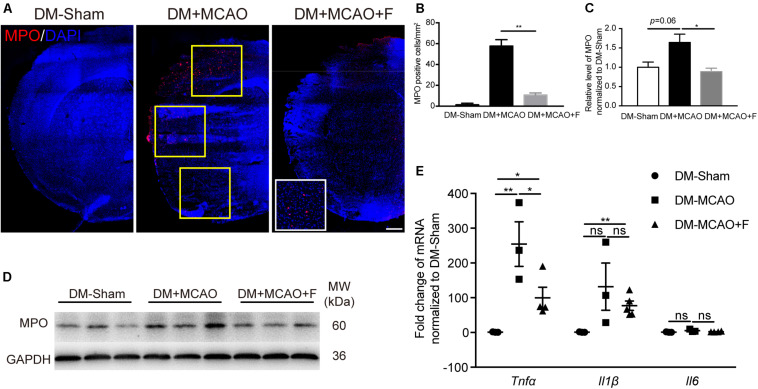
Fingolimod inhibited inflammatory response at 24 h after tMCAO. **(A)**, Micrographs of myeloperoxidase (MPO) and 4’,6-diamidino-2-phenylindole (DAPI) staining in the ischemic hemisphere of DM-Sham, DM + MCAO, and DM + MCAO + F groups, Scale bar, 500 μm. Yellow frames indicate fields for the MPO^+^ cell quantification. **(B)**, Bar graphs is the quantification of the number of MPO^+^ cells per square millimeter. **(C,D)**, MPO protein level, normalized to the sham group, from the western blot results. MW: molecular weight. **(E)**, The relative mRNA expression of *Tnf*α, *Il1*β, and *Il6* in brain tissues. Data are presented as mean ± SEM, *n* = 3/group, *p < 0.05, **p < 0.01.

The number of MPO positive cells was significantly reduced in the fingolimod treated group (Figures 4A,B; DM + MCAO + F vs. DM + MCAO, 10.67 ±2.028 vs. 57.67 ±6.119 cells, *p* < 0.01). The result of MPO protein quantification measured by western blot was consistent with the immunofluorescence staining result (Figures 4D,E; DM + MCAO + F vs. DM + MCAO, 0.2191 ±0.01325 vs. 0.4059 ±0.05285, *p* < 0.05). The mRNA levels of three inflammatory cytokines, *Tnf*α, *Il6*, and *Il1*β, were examined. We found that the mRNA of *Tnf*α was significantly reduced in the fingolimod-treated DM + MCAO + F group compared with the saline control DM + MCAO group (Figure 4C; DM + MCAO + F vs. DM + MCAO, 99.88 ±30.37 vs. 305 ±68.61, *p* < 0.05).

## Discussion

Approximately one-third of all stroke patients have diabetes (Benson and Sacco, 2000; Baird et al., 2002; Gray et al., 2004; Kaarisalo et al., 2005; Zsuga et al., 2012). It is therefore important to study stroke in the context of diabetes comorbidity. Although it has been reported that fingolimod was protective against ischemic stroke in animals without diabetes (Czech et al., 2009; Wei et al., 2011; Brait et al., 2016), as well as in a cohort of 11 stroke patients without diabetic comorbidity (Fu et al., 2014), to the best of our knowledge, our work is the first study to explore the effect of fingolimod treatment in a diabetic stroke model. We demonstrated that acute fingolimod treatment reduced the infiltration of MPO positive inflammatory cells and *Tnf*α gene expression at 24 h after tMCAO. However, acute fingolimod treatment significantly exacerbated BBB leakage and edema, counteracting its beneficial anti-inflammatory effects, subsequently resulted in unimproved outcomes. This work suggests that caution should be taken when considering using fingolimod in stroke patients with diabetes.

The effect of fingolimod on the outcome after ischemic stroke is a compounded result of multiple processes involved in post-stroke pathophysiology, such as inflammation, apoptosis, BBB disruption, acute excitotoxicity, glial activation, and polarization, angiogenesis, neurogenesis, and white matter injury and repair. Phosphorylated fingolimod, an analog of S1P, acts as an immunomodulator via binding to S1PR1 on lymphocytes and triggers its internalization and degradation, which results in reduced lymphocyte egressing from lymphoid tissues (Brinkmann et al., 2002; Matloubian et al., 2004). In our study, we observed that fingolimod inhibited the infiltration of inflammatory cells into the injured brain. It is consistent with the previous studies conducted in normal mice or *in vitro* models (Czech et al., 2009; Jackson et al., 2011; Wei et al., 2011; Nazari et al., 2016). It has been reported that fingolimod suppressed *Tnf*α at both the gene level and protein expression level detected by enzyme-linked immunosorbent assay (ELISA) *in vitro* and in a non-diabetic stroke mice model at 24 h and 3 days after tMCAO (Liesz et al., 2011; Wei et al., 2011; Noda et al., 2013). Our result is consistent with reported data that acute treatment with fingolimod suppressed *Tnf*α gene expression at 24 h after tMCAO. Studies showed that S1PR1 knockout or pharmacological inhibition of S1PR1 resulted in increased vascular permeability (Oo et al., 2011; Montrose et al., 2013; Christensen et al., 2016). It is consistent with what we observed in our experiments where fingolimod treatment reduced the expression of S1PR1 protein. It is known that diabetes worsens brain edema after ischemic injury (Kusaka et al., 2004). This might amplify the impact of fingolimod on BBB. As such, even though acute treatment with fingolimod was shown to be beneficial in normal mice after MCAO, such benefit was outweighed by its deleterious impact in BBB in diabetic animals in the time frame of this study. The experimental design and results of this work do not rule out the potential of acutely administrated fingolimod being beneficial in long-term recovery if we continue to follow the animals into the recovery phase. This warrants future investigation.

In addition, investigations on post-acute treatment of fingolimod should be studied further. It had been shown that fingolimod could reduce cell death of endothelial cells induced by inflammation (Spampinato et al., 2015). It was observed in other studies that vascular endothelial growth factor (VEGF) level was increased after fingolimod treatment (Brunkhorst et al., 2013), leading to subsequent angiogenesis. Moreover, a recent study demonstrated that enhanced angiogenesis was associated with microglial M2 polarization (Shang et al., 2020). Other studies also demonstrated that fingolimod could promote neurogenesis by enhancing the survival and proliferation of neural stem cells (Efstathopoulos et al., 2015; Sun et al., 2016; Tan et al., 2016; Jiang et al., 2017). The detailed molecular mechanisms by which fingolimod promote angiogenesis and neurogenesis warrants further study. Compared with normal rodents, diabetes animals show exacerbated cognitive deficits after stroke (Yatomi et al., 2015). Disruption of myelin integrity is closely associated with cognitive function. In a neonatal model of oxygen-toxicity, fingolimod ameliorated long-term cognitive deficits by reducing white matter damage (Serdar et al., 2016). It has been reported that fingolimod attenuated microglia-mediated neuroinflammation in the chronic ischemic model and promoted oligodendrogenesis via shifting microglia toward M2 polarization (Qin et al., 2017). Fingolimod also directly reduced the S1P receptor expression on microglia and prohibit microglia activation, promote remyelination consequently (Jackson et al., 2011; Airas et al., 2015). Studies with longer observation time might be needed to further assess the effect of fingolimod on oligodendrogenesis in the recovery phase. Given the significant negative impact of acute fingolimod treatment on BBB after diabetic stroke, it would also be of interest to test delayed treatment regimens with fingolimod.

## Conclusion

Our work revealed that acute treatment with fingolimod exacerbates BBB damage after cerebral ischemic injury in diabetic mice. Such deleterious effect of fingolimod on BBB integrity outweighs its beneficial effect of anti-inflammation, leading to unimproved endpoint outcomes.

## Data Availability Statement

All datasets presented in this study are included in the article/Supplementary Material.

## Ethics Statement

The animal study was reviewed and approved by the Institutional Animal Care and Use Committee of Shanghai Jiao Tong University, China.

## Author Contributions

WL designed and performed the experiments, analyzed the data, and drafted the manuscript. TH helped with animal surgery. LJ helped with experiment design and data analysis. RS did the animal behavior tests and revised the manuscript. YS, MM, and YM helped with the experimental operations and revised the manuscript. ZZ and YT discussed the results. G-YY and YW supervised the project design, data analysis and interpretation, and manuscript drafting and revision. All authors approved this manuscript.

## Conflict of Interest

The authors declare that the research was conducted in the absence of any commercial or financial relationships that could be construed as a potential conflict of interest.

## Abbreviations

BBB: blood–brain barrier
CD31: cluster of differentiation 31
CNS: central nervous system
IgG: Immunoglobulin G
MPO: myeloperoxidase
S1P: sphingosine-1-phosphate
S1PR1: sphingosine-1-phosphate receptor 1
tMCAO: transient middle cerebral artery occlusion
TUNEL: terminal-deoxynucleotidyl transferase mediated nick end label
ZO-1: zonula occludin1.
