# Correction: Fingolimod inhibits inflammation but exacerbates brain edema in the acute phases of cerebral ischemia in diabetic mice

**DOI:** 10.3389/fnins.2025.1639244

**Published:** 2025-06-19

**Authors:** Wanlu Li, Tingting He, Lu Jiang, Rubing Shi, Yaying Song, Muyassar Mamtilahun, Yuanyuan Ma, Zhijun Zhang, Yaohui Tang, Guo-Yuan Yang, Yongting Wang

**Affiliations:** ^1^School of Biomedical Engineering, Med-X Research Institute, Shanghai Jiao Tong University, Shanghai, China; ^2^Department of Neurology, Ruijin Hospital, School of Medicine, Shanghai Jiao Tong University, Shanghai, China; ^3^Department of Neurology, Zhongshan Hospital, Fudan University, Shanghai, China; ^4^Department of Neurology, Renji Hospital, School of Medicine, Shanghai Jiao Tong University, Shanghai, China

**Keywords:** diabetic stroke, diabetes mellitus, fingolimod, edema, inflammation

There was a mistake in Figure 3C as published. The GAPDH western blot image in Figure 3C was unintentionally incorrectly used. The corrected Figure 3C appears below.

**Figure 3:**
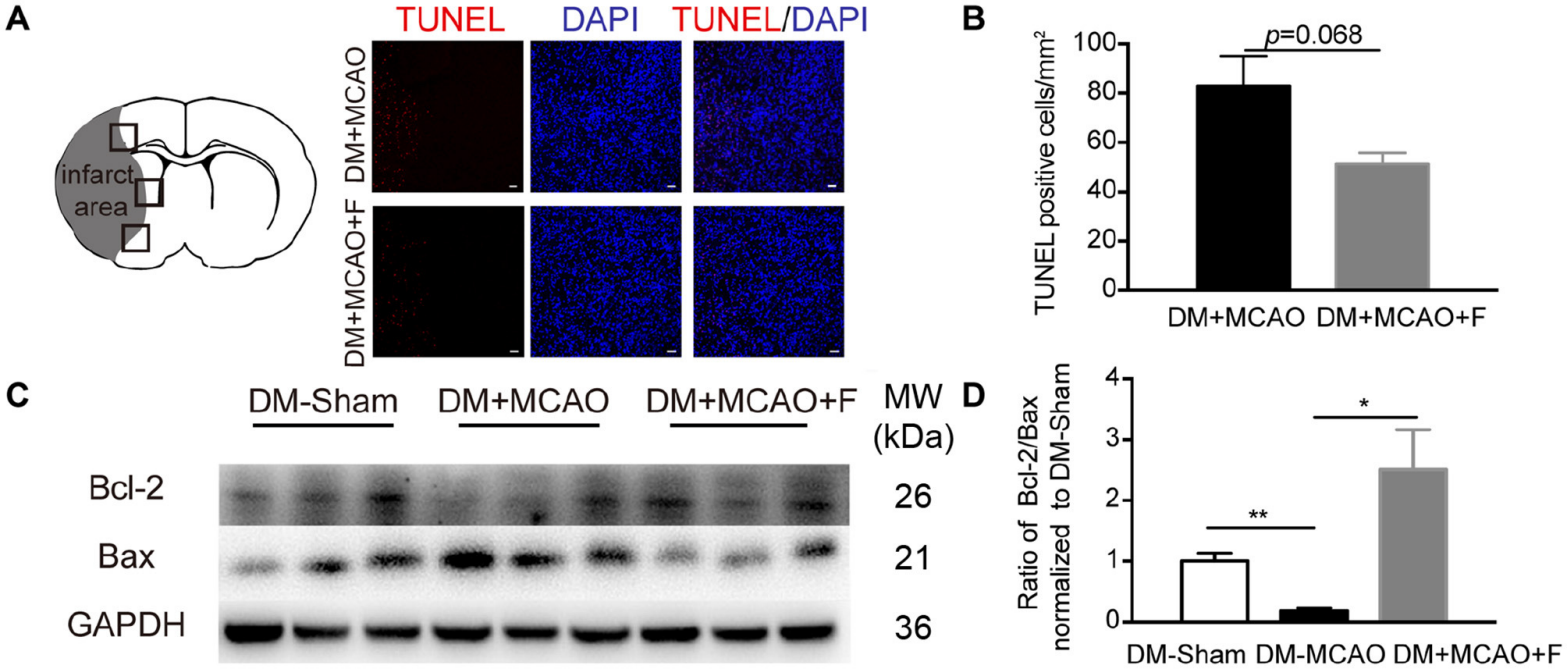
Fingolimod increased Bcl-2/Bax ratio but did not reduce the number of TUNEL + cells at 24 h after tMCAO. **(A)**, Micrographs of TUNEL staining in the peri-infarct region of DM + MCAO and DM + MCAO + F groups. Scale bar, 50 μm. Left panel showing the sketch of the brain section, boxes in it illustrating fields we are sampling in. **(B)**, Bar graph is the quantification of the number of TUNEL^+^ cells in DM + MCAO and DM + MCAO + F groups (*n* = 3/group). **(C)**, Western blot results of apoptotic factors Bcl-2 and Bax. **(D)**, Bar graph is the quantification of Bcl-2/Bax ratio based on Western blot data [*n* (Sham) = 3, *n* (DM + MCAO) = 3, *n* (DM + MCAO + F) = 6]. MW, molecular weight. Data are presented as mean ± SEM, *p < 0.05, **p < 0.01.

In the published article, there was an error in citing data within the Results section.

A correction has been made to Results, *Acute Treatment with Fingolimod Failed to Improve Endpoint Outcomes at 24 h After tMCAO in Diabetic Mice*, Paragraph 2. This sentence previously stated:

“No significant difference in neurological score (DM + MCAO vs. DM + MCAO + F, 12.40 ±3.29 vs. 15.42 ±4.12) (Figure 1E) or brain infarction volume (DM + MCAO vs. DM + MCAO + F, 62.81 ±3.15 mm^3^ vs. 71.22 ±3.98 mm^3^) (Figures 1F–G) was observed.”

The corrected sentence appears below:

“No significant difference in neurological score (DM + MCAO vs. DM + MCAO + F, 12.40 ±3.29 vs. 15.42 ± 4.12) (Figure 1E) or brain infarction volume (DM + MCAO vs. DM + MCAO + F, 40.7 ± 8.90 mm^3^ vs. 34.4 ± 6.97 mm^3^) (Figures 1F–G) was observed.”

The authors apologize for this error and state that this does not change the scientific conclusions of the article in any way. The original article has been updated.

