# Supplementary material for: Fingolimod Inhibits Inflammation but Exacerbates Brain Edema in the Acute Phases of Cerebral Ischemia in Diabetic Mice
Source: Front Neurosci. 2020 Aug 11;14:842. doi: 10.3389/fnins.2020.00842 (PMC7432267; doi:10.3389/fnins.2020.00842)
Supplement: Supplementary file 1 [file Data_Sheet_1.docx]

**Supplementary Material:**

Fingolimod Inhibits Inflammation but Exacerbates Brain Edema in the Acute Phases of Cerebral Ischemia in Diabetic Mice

**Wanlu Li^1^, Tingting He^2,3^, Lu Jiang^1^, Rubing Shi^1^, Yaying Song^2,4^, Muyassar Mamtilahun^1^, Yuanyuan Ma^2,3^, Zhijun Zhang^1^, Yaohui Tang^1^, Guo-Yuan Yang^1,2^**^*^**, and Yongting Wang^1^**^*^

**Methods**

**Induction of diabetes mellitus**

Diabetes mellitus was induced by intraperitoneally injecting a single dose of STZ (150 mg/kg) to 8-hour-fasted animals. STZ powder was dissolved in 0.1 M sodium citrate buffer (pH 4.5) to a final concentration of 10 mg/ml. At 0, 7, and 14 or 28 days after STZ injection, the serum glucose level of the mice was measured using the glucose oxidase assay kit (Bayer Health Care, Leverkusen, Germany). Mice with serum glucose level under 16.7 mmol/L were excluded from further experiments. tMCAO surgery was carried out at 28 days after STZ injection for the acute treatment regimen study.

**Transient middle cerebral artery occlusion**

Transient middle cerebral artery occlusion was performed as previously described. In brief, adult male ICR mice were anesthetized with 1.5% isoflurane, and a 30% O_2_/68.5% NO mixture and animals were put on a heating pad (RWD Life Science, Shenzhen, China) throughout the surgery to maintain a body temperature of 37 ºC. A 6-0 suture (Covidien, Mansfield, MA) coated with silicon was gently inserted into the middle cerebral artery through an incision of the external carotid artery. The suture was withdrawn after 1 hour to allow reperfusion. Cerebral blood flow was measured with laser Doppler flowmetry (Moor Instruments, Devon, UK) before surgery, after occlusion, and after reperfusion to ensure accurate model control. Successful reperfusion is defined as the CBF restored to 80% of the baseline after the suture was withdrawn.

**Assessment of brain lesion size**

Brain infarct volume and edema were assessed based on cresyl violet staining. Mice were perfused intracardially with normal saline after euthanizing and followed by fixating with 4% paraformaldehyde (PFA, Sinopharm Chemical Reagent, Shanghai, China). After perfusion, the removed brains were frozen rapidly in −40ºC isopentane (Sinopharm Chemical Reagent, Shanghai, China), and then the frozen tissue was cut into a series of 20-μm coronal sections from 1.3 to -2.7 mm of the bregma using a cryostat (Leica, Solms, Germany). A total of 20 coronal sections spanning the region from 1.3 to -2.7 mm of the bregma were collected to evaluate brain lesion size. The area of these sections was measured by ImageJ software (National Institutes of Health, Bethesda, MD). The results were calculated using the following formulas:

$$V(infarct)=\sum\frac{h}{3}\left[ {\Delta S}_{n}+\sqrt{\Delta S_{n}\times\Delta S_{n+1}}+\Delta S_{n+1} \right]$$

$${\Delta S}_{n}=S_{n_{Contl}}-(S_{n_{Ipsil}}-S_{n_{infarct}})$$

$${\Delta S}_{n+1}=S_{{n+1}_{Contl}}-(S_{{n+1}_{Ipsil}}-S_{{n+1}_{infarct}})$$

$$edema percentage (\%)=\left[ \sum\left( 1-{(S}_{n_{Ipsil}}-S_{n_{infarct}})/S_{n_{Contl}} \right)/n \right]\times100$$

h indicates the distance between two adjacent coronal sections, h=200μm.

**Western blot analysis**

Mice were sacrificed under deep anesthesia at 24 hours after tMCAO. Brains were quickly removed and cut into four 2-mm-thick sections around the infarction region. The second section of the ipsilateral hemisphere was used to extract proteins. Western blot was performed as previously described. Forty micrograms extracted protein was loaded into SDS-PAGE gel for electrophoresis. The proteins in gels were transferred onto a polyvinylidene fluoride membrane. After blocking with milk, the membrane was incubated in the primary antibody at 4°C overnight and then incubated with HRP-conjugated secondary antibody for 2 hours at room temperature. The membrane was then developed by reacting with an enhanced chemiluminescence substrate (Pierce, Rockford, IL) according to the manufacturer’s protocol. The primary antibodies were MPO, ZO-1, Occludin, S1PR1, Bcl2, Bax, GAPDH. The results were recorded with an imaging system (Bio-Rad, Hercules, CA) and analyzed with ImageJ software.

**Real-time quantitative PCR**

Mice were sacrificed under deep anesthesia at 24 hours after tMCAO. Brains were quickly removed and sectioned into four 2-mm-thick sections. The third section of the ipsilateral hemisphere was used to extract RNA. The cortex and striatum in each hemisphere are separated. RT qPCR procedure was carried out as previously described. We used the TRIzol reagent (Invitrogen) to extract RNA first, followed by synthesizing the cDNA with reverse transcription. The reaction condition of RT qPCR was chosen according to the protocol of the SYBR Premix Ex Taq Kit (Takara, Dalian, China). The expression of mRNA was calculated with 2^-∆CT^ method. ∆CT indicates the difference between CT values of target genes and CT values of the *Gapdh* gene.

**Table 1. Body weight and blood glucose**

|  | Normal mice (n=15) | | Diabetic mice (n=70) | |
| --- | --- | --- | --- | --- |
| Time after STZ injection (days) | Body weight (g)+SEM | Blood glucose (mmol/L)+SEM | Body weight (g)+SEM | Blood glucose (mmol/L)+SEM |
| -28 | 21.67+0.24 | 9.8+0.16 | 22.99+0.16 | 9.87+0.30 |
| -21 | 27.25+0.69 | 9.32+0.27 | 23.57+0.46 | 30.94+1.20 |
| 0 | 32.97+1.62 | 9.50+0.24 | 25.59+1.11 | 32.76+0.66 |

**Table 2. Clark neurological score (0-28)**

|  | 0 | 1 | 2 | 3 | 4 |
| --- | --- | --- | --- | --- | --- |
| Body symmetry | Normal | Slight asymmetry | Moderate asymmetry | Prominent asymmetry | Extreme asymmetry |
| Gait | Normal | Stiff, inflexible | Limping | Trembling, drifting, falling | Does not walk |
| Climbing on 45° angle | Normal | Climbs with strain, limb weakness | Holds onto slope, doesn’t slip or climb | Slides down slope, unsuccessful effort to prevent fall | Slides immediately, no effort to prevent fall |
| Circling behavior | Not present | Predominantly 1-sided turns | Circles to 1 side | Circles constantly to 1 side | Pivoting, swaying, or no movement |
| Front limb symmetry (mouse suspended by its tail) | Normal | Light asymmetry | Marked asymmetry | Prominent asymmetry | Slight asymmetry, no body/limb movement |
| Compulsory circling (front limbs in bench, rear suspended by tail) | Not present | Tendency to turn to 1 side | Circles to 1 side | Pivots to 1 side sluggishly | Does not advance |
| Whisker response | Symmetrical response | Light asymmetry | Prominent asymmetry | Absent response ipsilaterally, diminished contralaterally | Absent proprioceptive response bilaterally |

**Table 3. The primers sequence**

| Gene | Forward primer | Reverse primer |
| --- | --- | --- |
| *Tnfα* | TTGGAGTCATTGCTCTGTGA | GGGTCAGAGTAAAGGGGTCAG |
| *Il1β* | CTCTCCACCTCAATGGACAGA | TTTTGTCGTTGCTTGGTTCTC |
| *Il6* | ATGGATGCTACCAAACTGGAT | TGAAGGACTCTGGCTTTGTCT |
| *Zo1* | ACAGCTACAGGAAAATGACCGA | GCTCAGGATCTGGGTGACTT |
| *Occludin* | AGATTCCTCTGACCTTGAGTGTGG | TCCTGCTTTCCCCTTCGTG |
| *S1pr1* | ATGGTGTCCACTAGCATCCC | CGATGTTCAACTTGCCTGTGTAG |
